# Tear Proteomics Approach to Distinguishing Primary from Secondary Sjögren’s Syndrome for Dry Eye Patients with Long-Term Instillation of Eyedrops

**DOI:** 10.3390/ijms232315239

**Published:** 2022-12-03

**Authors:** Yu-Ting Hsiao, Yu-Ting Huang, Hun-Ju Yu, Po-Chiung Fang, Ming-Tse Kuo

**Affiliations:** 1 Department of Ophthalmology, Kaohsiung Chang Gung Memorial Hospital and Chang Gung University College of Medicine, Kaohsiung 83301, Taiwan; 2 School of Medicine, Chang Gung University, Taoyuan City 33302, Taiwan

**Keywords:** dry eye disease, biomarkers, Sjögren syndrome, tear fluids, proteomics

## Abstract

The diagnosis and monitoring of Sjögren syndrome (SS) is often difficult, requiring a multidisciplinary approach with invasive procedures. Our aim is to elucidate the tear protein alterations of dry eye disease (DED) with primary SS (pSS) and secondary SS (sSS) with the long-term instillation of eyedrops. We collected clinical demographics and tear fluid (TF) samples from DED patients with no autoimmune diseases (non-SS-DED), pSS-DED, and sSS-DED patients, followed by TF screening with tandem mass tagging-labeling gel-free proteomics assay. Bioinformatic analysis via Ingenuity Pathway Analysis was used to identify functional pathways and interacting networks. Validation of candidate proteins with enzyme-linked immunosorbent assay on the tear samples was done. The top functional pathways of the two comparisons (sSS-DED vs. pSS-DED and sSS-DED vs. non-SS-DED) were both associated with inflammation and stress-related signaling. After constructing an interaction network model with the selected candidate proteins, five proteins were identified. A Disintegrin and Metalloproteinase domain-containing protein 10 (ADAM10) was found to be an important candidate biomarker in all groups, followed by epidermal growth factor (EGF) in TF. This study revealed novel DED markers, ADAM10 and EGF, in differentiating between primary and secondary SS patients from tears by in-depth proteomic analysis.

## 1. Introduction

Dry eye disease (DED) is characterized by homeostatic loss of the tear film, which can cause ocular discomfort and visual disturbance [1]. Despite DED being a common and multifactorial ocular surface disorder, there are still significant differences in pathophysiological mechanisms in these patients with ocular and systemic diseases [2]. Sjögren’s syndrome (SS) is an autoimmune disorder that affects secretory glands, causing dry eyes and mouth, particularly in women over the age of forty. Primary SS (pSS) is defined when the patient has no other connective tissue disease, while secondary SS (sSS) is diagnosed if the patient has an underlying connective tissue disorder [3]. Due to the fact that there is currently no definite cure for SS, current treatments are limited to only managing SS symptoms rather than eliminating them [4].

The diagnosis of SS is often difficult as there is no single diagnostic gold standard test, and a multidisciplinary approach (including rheumatology, ophthalmology and oral health experts) with invasive procedures are required for the diagnosis of SS [5,6]. Differentiation between pSS and SS from systemic lupus erythematosus (SLE), rheumatoid arthritis (RA), and other collagen vascular diseases can be challenging, since all of these conditions may initially present with nonspecific manifestations including arthralgias, myalgias, and low-grade fever [7]. However, there are few studies on the etiological subcategories of DED in patients with or without systemic autoimmune disease.

Corneal ulceration and corneal melting, which may lead to corneal perforation, are rare yet severe complications of established SS, usually secondary to DED patients with underlying connective tissue disorders [8]. In RA-related DED patients who develop ulcerative keratitis, immediate combination therapy including corticosteroids and calcineurin inhibitors, as well as biologic disease-modifying antirheumatic drugs and possible corneal transplantation is essential [9]. It is challenging to differentiate between pSS and sSS from ocular findings, and thus we are prompted to discover biomarkers for a personalized approach into identifying patients with other connective tissue disorders.

Proteomic analysis of tear fluids (TF) has emerged as one of the most promising approaches for DED biomarkers investigations [10,11]. The composition of the tear film is made up of a variety of proteins, including enzymes, growth factors, cytokines, neuropeptides, and also other substance classes including lipids, carbohydrates and salt [12]. Tears can be easily collected using minimally invasive techniques; therefore, its analysis is a relevant method for developing new ocular surface disease diagnoses and treatment alternatives. Tear proteins have been investigated intensively in ocular diseases such as glaucoma, dry eye, keratoconus, diabetes mellitus or cancer [2,13]. In our previous study, we found tear proteomics to be a promising method for monitoring SS-DED patients. The concentration ratio of tear matrix metallopeptidase 9/lactoferrin correlated significantly and positively with ocular surface inflammation in SS-DED subjects [14]. The high concentration of proteins and easy acquisition of tear fluids have made tears a sought-after target for proteomics for precision medicine in ophthalmology research [15].

In spite of recent progress in our knowledge of SS and its pathogenesis, there is still a lack of insight upon the interplay between these primary and secondary SS patients’ tears components [4]. Effective monitoring of DED and early diagnosis of SS patients underlying connective tissue diseases may prevent serious corneal complications, which are difficult to treat. The aim of our study is to compare the tear proteomic profile of dry eye patients with and without autoimmune diseases and explore possible biomarkers.

## 2. Results

### 2.1. Subject Characteristics

A total of 36 subjects with DED were included in the analysis, with 12 subjects in each group. The comparisons of demographic data are shown in Table 1. Significant differences were found in Oxford staining score (*p* = 0.01) and ocular surface redness over the limbal nasal area (*p* = 0.041). Post-hoc analysis of limbal nasal redness revealed significance between sSS-DED and pSS-DED. Upon comparing from DED patients with no autoimmune diseases (non-SS-DED), pSS-DED, to sSS-DED, Ocular Surface Disease Index (OSDI), the measurement period of non-invasive keratograph break-up time (NIKBUT), and average NIKBUT showed statistical significance in a linear trend.

### 2.2. Proteome Profiling of TF

The proteins in pooled TF samples from patients with non-SS-DED (n = 12), pSS-DED (n = 12), and sSS-DED (n = 12) were analyzed by tandem mass tagging (TMT) mass spectrometry. In total, over 2000 proteins were identified in the TF. Upon comparing sSS-DED and non-SS-DED groups, we found 209 differentially expressed proteins (DEPs), including 126 upregulated and 83 downregulated proteins. When comparing between sSS-DED and pSS-DED groups, a total of 89 DEPs were identified, with 23 upregulated and 66 downregulated proteins.

### 2.3. Gene Ontology Analysis in the TF of DED Patients

**Figure 1:**
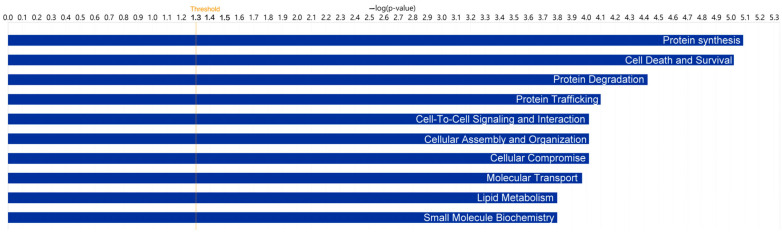
A functional analysis of the differentially expressed proteins with Ingenuity Pathway Analysis in sSS-DED patients versus pSS-DED patients indicated its top association with protein synthesis.

**Figure 2:**
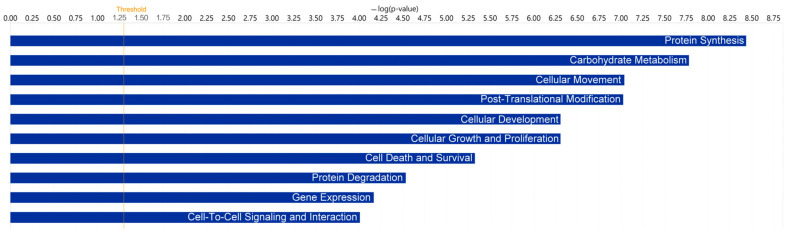
A functional analysis of the differentially expressed proteins with Ingenuity Pathway Analysis in sSS-DED patients versus non-SS-DED patients indicated its top association with protein synthesis.

To determine the functional roles associated with the proteins in TF of DED patients, we compared the DEPs using the functional analysis and canonical pathway features in Ingenuity Pathway Analysis (IPA). The top categories are presented in Table 2 and Table 3, Figure 1 and Figure 2.

Notably, acute phase response signaling was the top canonical pathway in the sSS-DED vs. pSS-DED group (Table 2), whereas EIF2 signaling was in the top category in the sSS-DED vs. non-SS-DED comparison (Table 3). Both were associated with inflammation and stress-related signaling. Moreover, functional analysis of the DEPs resulted in protein synthesis with the top association in TF of both comparisons of DED patients (Figure 1 and Figure 2).

### 2.4. Interaction Network Model of Candidate Proteins Revealed a Key Regulator in DED

**Figure 3:**
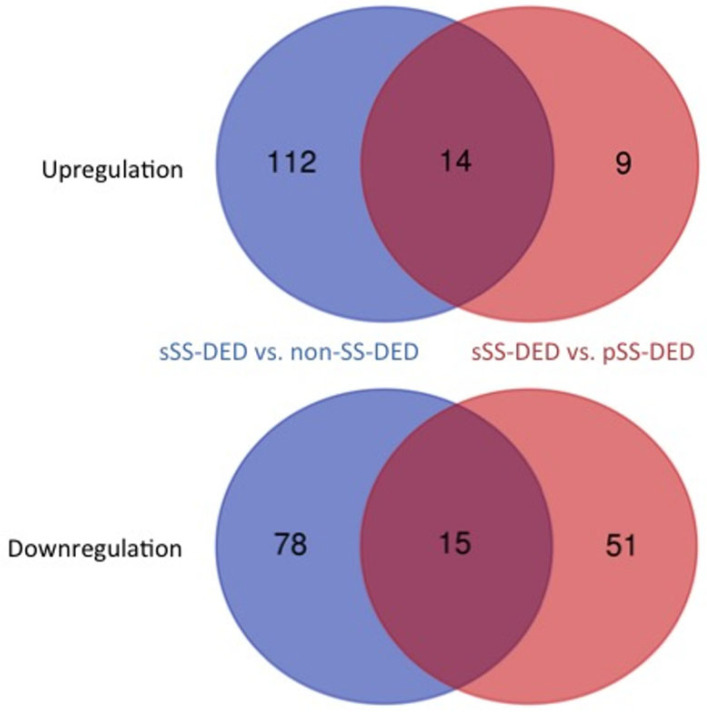
The candidate proteins were those overlapping with differentially expressed proteins in sSS-DED patients versus non-SS-DED and pSS-DED, respectively. Twenty-nine proteins (14 upregulated and 15 downregulated) were identified.

**Figure 4:**
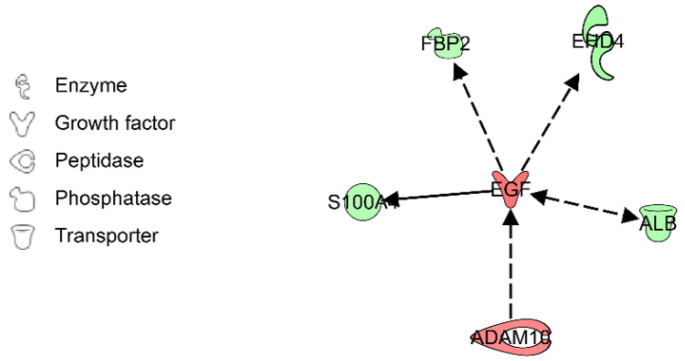
Connections and interactions identified between the 29 candidate proteins using Ingenuity Pathway Analysis. Individual nodes are hidden.

In the DEPs of both comparisons, 14 upregulated and 15 downregulated proteins showed the same increasing or decreasing expression patterns (Figure 3). We constructed an interaction network model with these 29 candidate proteins, using IPA. As a result, 4 proteins, including albumin (ALB), A Disintegrin and Metalloproteinase domain-containing protein 10 (ADAM10), S100 calcium binding protein A4 (S100A4), fructose-1,6-bisphosphatase isozyme 2 (FBP2), and EH domain-containing protein 4 (EHD4), were all found to form a close network with epidermal growth factor (EGF) (Figure 4).

### 2.5. Verification of Candidate Biomarkers

We performed an enzyme-linked immunosorbent assay (ELISA) analysis using 1 μL tear sample from each DED patient to verify four candidate proteins from the proteomics data. As a result, ADAM10 was confirmed to be consistent with the alterations in the proteomics data, which not only showed significance upon comparing among the three groups (*p* = 0.018), but also resulted in a significant linear trend (*p* = 0.011). After Tukey correction, both comparisons revealed significance (sSS-DED vs. non-SS-DED, *p* = 0.028; sSS-DED vs. pSS-DED, *p* = 0.043). Based on ELISA quantification, EGF also showed significance when comparing among the three groups (*p* = 0.023), with significance found between sSS-DED and pSS-DED (*p* = 0.02) after the post-hoc test (Table 4).

## 3. Discussion

In this pilot comparative study, we evaluated the tear film of three patient subgroups all with dry eye disease—those without autoimmune disease, with pSS, and with sSS, using TMT labeling proteomics investigation. Despite the differences found among the tear proteome of DED patients with or without systemic conditions, we found ADAM10 to be a vital candidate biomarker in all groups.

Ocular surface disease is a mean feature in both patient’s clinical characteristics and proteome profile. Our study found that the Oxford staining score and limbal nasal ocular surface redness showed significant changes among the three groups. Clinical parameters such as OSDI and total measurement period of NIKBUT were also significant in the linear trend (Table 1). Canonical pathway analysis of tear proteins revealed pathways correlated with stress-related signaling and inflammation in both comparison groups (Table 2 and Table 3). It is known that immune response-related proteins are considered meaningful biomarkers in TF [16]. Previously, TF from pSS patients demonstrated the involvement of innate and adaptive immune systems, when comparing with non-SS DED subjects [17]. Ours is the first to compare the protein alterations between the TF of sSS and pSS patients.

The differential diagnosis between pSS and sSS is critical to ensure timely and accurate treatment of the complications associated with the disease such as ulcerative keratitis [7]. In patients with autoimmune disease with SS, DED is a common complication [18]. Our results showed that tear meniscus height (TMH) was lower in pSS patients than those with sSS (Table 1), which is consistent with the findings of previous studies of DED patients with autoimmune diseases [19,20]. Primary SS patients often present with more severe aqueous deficiency [21], and it was suggested that different extents in lacrimal gland damage and meibomian gland dysfunction (MGD) in SLE patients may contribute to the severity of DED [20].

ADAM10 was found to be a crucial biomarker in both pSS and sSS DED patients in this study. The protein levels increased significantly from non-SS patients, to pSS-DED and sSS-DED subjects (Table 4). In limbal-corneal epithelial compartmentalization, direct migration of EphA2-expressing cells depended on ADAM10 and epidermal growth factor receptor (EGFR) signaling pathway [22]. ADAM10 has also been identified in a corneal model of epithelial resurfacing [23]. Immune responses may lead to tissue damage, followed by wound repair [24]. According to the Oxford staining score of our patients, which is a scheme used to estimate surface damage in dry eye, results showed a significantly increasing proportion of patients with more severe grading from the non-SS-DED group to the sSS-DED group (Table 1). Furthermore, functional analysis of DEPs revealed protein synthesis as the top association in both comparison groups. The increased cytokine secretion by the corneal epithelium or a concomitant ocular surface disease condition may be responsible for these tear proteome alterations in certain DED patients [16].

Additionally, ADAM10 has also been linked to a variety of human disorders, including neurodegeneration, immune system dysfunction, and cancer [25]. The protease modulates the process of leukocyte recruitment during inflammation, and is also able to mediate the release of TNF-α and IL-6R [26]. Evidence regarding the role of ADAM10 inhibitors in RA and SLE indicates that it may be a useful therapeutic target and also provide a personalized approach for predicting autoimmune disease patients’ responsiveness to biologic therapies [27]. ADAM10 was found to be a biomarker for predicting patient responsiveness to biologic therapies by drawing the patients’ blood. Our study is the first to report that ADAM10 may also be a candidate biomarker for differentiating DED patients with or without autoimmune diseases through tear collection, which is a relatively non-invasive method.

EGF, a cytokine produced by the lacrimal glands, is one of the most prominent growth factors in human tears. EGF is essential for promoting wound epithelial healing and maintaining homeostasis of the ocular surface epithelium [28]. However, when tear EGF concentration rises above a certain level, it may induce corneal and meibomian gland (MG) ductal epithelium hypertrophy [29]. It was reported that EGF concentration in tears was highest in DED patients with MGD, corneal epithelial fibrosis, and MG orifice metaplasia [29,30]. Tear EGF was found to be lower in DED subjects with SS than those without [29]. Similarly, our ELISA verifications of the three DED subgroups revealed pSS-DED with the lowest EGF concentrations (Table 4). The variations in TF EGF levels in sSS-DED patients may be a consequence of systemic medication usage or underlying disease stability. It is hypothesized that DED subgroups with corneal fibrotic changes and neovascularization may be the cause of higher tear EGF concentrations.

Previous proteomics studies have also investigated additional biomarkers in SS patients. In pSS patients versus controls, protein metabolism was found to be a significantly upregulated biological process, involving MMP8, SERPINB5, RPLP2, CSTB, and CST3, etc. [24]. Our results also showed protein synthesis as the top associated functional pathway according to IPA analysis. Another research characterization, the proteomic profiling of SS, found that the most upregulated cellular processes in the pSS group when compared to the healthy controls was retina homeostasis, followed by other central innate and adaptive immune responses. Although they found that erythrocyte band 7 integral membrane protein (STOM), Annexin A4 (ANXA4), and Annexin A11 (ANXA11) to be expressed significantly more in the pSS group when compared to healthy controls, they found no proteins to be significantly different when comparing non-SS subjects to pSS participants [17].

A limitation of this study was the small number of cohort samples. Future studies of larger patient cohorts are needed to apply these biomarkers in diagnosis. However, the pilot study showed promising results, narrowing the complex tear proteome into some potential molecules for large-scale research. Although the cost of using proteomics as the main diagnostic method should be considered, research and development in precision medicine are often costly. Nonetheless, prior to clinical utilization, an understanding of the pathophysiological and biochemical pathways is needed. Also, future studies, including additional repeatability and validation assessments, should be considered.

## 4. Materials and Methods

### 4.1. Enrollment of Patients and Assessment of Ocular Surface Dryness

This cross-sectional, case control prospective study was conducted at the Cornea Division of Kaohsiung Chang Gung Memorial Hospital between December 2019 and June 2022. All procedures complied with the tenets of the Declaration of Helsinki. Institutional Review Board/Ethics Committee approval was obtained from the Committee of Medical Ethics and Human Experiments of CGMH, Taiwan (IRB approval no. 201900954B0), and informed consent was obtained from all patients. DED was diagnosed according to an OSDI score ≥ 13, and positive for at least one of the following: TMH < 0.20 mm or NIKBUT first <6 s. Subjects were included if they had been instilling lubricants for DED for one year or more. Patients were excluded if they had acute inflammatory eye diseases in the recent half-year, received ocular or eyelid surgeries within six months, or were pregnant. The initial diagnosis was consistent with the American Rheumatology Association’s criteria for RA and SLE [31,32]. Clinical tests for DED and human fluid-collection of TF were carried out by masked technicians and ophthalmologists, respectively.

### 4.2. Assessment Protocol

Following the completion of a dry eye questionnaire, OSDI [20], a masked examiner evaluated each patient’s binocular ocular surface homeostasis. Each subject was examined in the following order: TMH, ocular surface redness scan (R-scan), and NIKBUT. Finally, to assess the corneal staining using the Oxford scheme, the cornea was stained with fluorescence and surveyed under a blue-light illuminating slit-lamp [33].

### 4.3. Meniscometry for Determining Tear Volume

Tear volume was quantified using TMH. The Keratograph 5M (K5M; OCULUS, Optikgerate GmbH, Wetzlar, Germany) was used to capture three photographs of the anterior eye with infrared illumination of 880 nm wavelength [34]. The TMH was measured as the distance along a vertical axis spanning from the lower lid margin to the top of the inferior meniscus. Measurements were made as close to the center of the eyelid as possible, and the mean values of the three photographs of each subject were recorded [14].

### 4.4. Quantification of Ocular Surface Redness

The K5M tear film analyzer was used for R-scan evaluation [35,36]. After blinking, subjects were instructed to focus on the mark inside the camera. The 22-mire Placido ring system would then reflect on the entire cornea, and the analyzer could detect the conjunctival blood vessels and quantify the severity of redness with built-in software. Within 10 s, the index of mean bulbar redness score was procured.

### 4.5. Evaluation of Tear Film Stability

NIKBUT was measured using the K5M analyzer’s tear film surface quality break-up time [37]. All subjects were directed to look straight forward, blink twice, and then keep their eyes open as long as they could. Three parameters were automatically generated after completing the exam: (1) NIKBUT first: the time when the first distortion in the reflected Placido ring occurs; (2) NIKBUT avg: associated with the localized tear break-up times and calculated based on the average time of all observed perturbations; (3) measuring time: tolerable duration of the NIKBUT test, the interval between the second blink that began the recording and the subsequent blink that signaled the completion of the test.

### 4.6. Sampling of Tear Fluid for Proteomics and ELISA

After the above examinations, each patient was instructed to lie down in a supine position on an operation table for tear collection. Without using topic anesthesia, tear fluid samples were obtained using a standardized eye-flush procedure [38]. A 60 μL drop of non-preserved normal saline was instilled on the cornea by a physician, with gentle eyelid support using the forefinger and the thumb. After that, the subject was told to keep their eye open, shift it around, and then gaze nasally while leaning their head to one side by roughly 10–15 degrees. A 50 μL tear sample was obtained by collecting tears that had gathered at the fornix near the lateral canthus using an automated pipette (Pipetty 0.1–20 μL, Icomes Lab Co. Ltd., Iwate, Japan) under a surgical biomicroscope.

As soon as the tear samples were collected, they were promptly centrifugated at 3000× *g* in a microcentrifuge tube for 10 min at 4 °C, and the 40 mL supernatant fluid was then frozen at −20 °C. After tear samples of all subjects were collected, the total protein of each tear sample was thawed to 0–4 °C and quantified using the Bradford protein-binding assay (Bio-Rad Protein Assay, Bio-Rad Laboratories Taiwan Ltd., Taipei, Taiwan). One part of each tear sample was aspirated for proteomic analysis. The volume of the tear sample was adjusted to ensure that each sample had the same amount of total protein, 5 μg, for proteomic analysis, then mixed in groups of six. After quantifying the mixed tear samples, 25 μg from each mixed sample was extracted. The residual part of each tear sample was serially diluted with normal saline to meet the detection range of different ELISA kits for determining the concentrations of each target protein. The tear concentration of each protein was finally obtained after multiplying each detected concentration by its dilution factor, respectively.

### 4.7. TMT Gel-Free Proteomics to Globally Screen Serum Proteins

To globally screen proteins in tear fluid, we conducted TMT gel-free proteomics assay. First of all, we selected ten tear fluid samples and had them subjected to protein digestion by trypsin enzyme to generate peptides. Then, the ten peptide samples were subjected to peptide labeling with the TMT 10-plex Reagents Kit (90111, Thermo Fisher, Waltham, MA, USA) by referring to the manufacturer’s protocols. Next, the labeled peptide samples, passing the standard QC check, were analyzed with LC/Q-Exactive Orbitrap MS (Thermo Fisher) for 24 h. The generated raw data was further analyzed with Proteome Discoverer v2.4 (Thermo Fisher) by referring to the MASCOT 2.5 database (Matrix science). By doing so, the relative abundances of proteins in tear fluid were detected. The proteins, with false discovery rates less than 1% and with at least two unique peptides, were considered. In addition, median normalization was applied to normalized the protein abundances among samples for reducing the system error which may be contributed by sample preparation.

### 4.8. Analysis for Proteomics Data

Additional statistical and bioinformatics analyses were carried out using Partek software. DEPs were those with expression levels greater than the ±1.5-fold change and *p* < 0.05 from Student’s *t*-test. The resulting significant DEPs were analyzed with further annotation enrichment analyses.

### 4.9. Enrichment Analysis Using Gene Ontology and Network Analysis

A gene ontology search using IPA v9.0 (Ingenuity Systems, Redwood City, CA, USA) was performed to explore the protein networks and functional pathways of the proteins differentially expressed in TF of DED patients. UniProt IDs are mapped by IPA into the ingenuity knowledge base. The top pathways were identified in sSS-DED vs. pSS-DED and sSS-DED vs. non-SS-DED groups. Candidate proteins were identified as those with simultaneous upregulated and downregulated patterns in both comparisons. To construct a network model for candidate proteins, we identified interactions between the proteins also using IPA.

### 4.10. ELISA Validation for Selected Candidate Proteins

Among the candidate proteins detected by TMT-labeling proteomics analysis, we selected four potential proteins as follows: EGF, ADAM10, ALB, and S100A8, for further ELISA validation in all three groups. The procedure for ELISA was performed according to the manufacturer’s instructions.

### 4.11. Statistical Analysis

In descriptive analyses, quantitative variables were shown as mean ± standard deviation, and categorical data were represented as numbers and percentages. Continuous variables relating to participant demographics and clinical parameters between the groups were compared using one-way ANOVA. The post-hoc analyses (Tukey’s Post Hoc Test with 95% confidence interval) tested the differences and linear trends between groups, whereas Fisher’s exact or Pearson’s chi-squared (χ^2^) tests were used to compare categorical data. A *p* value of <0.05 was considered to be significant. Statistical analysis was performed using GraphPad Prism version 9.4.1.681 for Windows (GraphPad Software, San Diego, CA, USA).

## 5. Conclusions

Our study provides essential information regarding biomarker candidates for SS-DED patients with and without rheumatic disorders from the human ocular surface via a proteomics approach and subsequent ELISA verification. Identifying ADAM10 in these subgroups of patients may lead to more accurate diagnosis and ultimately to developing targeted drug therapies for SS-DED in precision medicine. Further researches are required to determine the clinical application of the tear biomarker in the future.

## Figures and Tables

**Table 1:** Demographic data.

	Non-SS-DED (n = 12)	pSS-DED (n = 12)	sSS-DED (n = 12)	*p* Value	*p* Value (Trend) *^a^*
Age	60.75 ± 1.77	63.58 ± 1.38	59.92 ± 2.89	0.446	0.782
OSDI ^*b*^	40.38 ± 20.20	50.24 ± 29.97	64.93 ± 27.06	0.103	0.036
Oxford staining score				0.010	0.003
0	11 (91.7)	5 (41.7)	3 (25.0)		
1	0	4 (33.3)	2 (16.7)		
2	1 (8.3)	1 (8.3)	4 (33.3)		
4	0	2 (16.7)	3 (25.0)		
Tear meniscus height	0.23 ± 0.10	0.16 ± 0.08	0.31 ± 0.33	0.209	0.337
Noninvasive tear breakup time					
Measurement period	15.74 ± 8.50	12.13 ± 7.77	9.74 ± 4.62	0.135	0.048
NIKBUT First ^*c*^	9.27 ± 7.50	8.69 ± 6.46	5.62 ± 2.37	0.279	0.137
NIKBUT Avg ^*d*^	11.69 ± 7.26	10.21 ± 6.25	6.85 ± 3.16	0.130	0.050
Ocular surface redness					
Bulbar redness	1.25 ± 0.41	1.49 ± 0.54	1.57 ± 0.68	0.357	0.172
Area	8.78 ± 4.65	8.19 ± 3.75	8.07 ± 4.17	0.906	0.679
Bulbar temporal	1.21 ± 0.40	1.58 ± 0.53	1.55 ± 0.67	0.189	0.133
Bulbar nasal	1.38 ± 0.65	1.41 ± 0.64	1.66 ± 0.75	0.545	0.316
Limbal temporal	0.83 ± 0.41	1.10 ± 0.44	1.18 ± 0.68	0.244	0.110
Limbal nasal	0.83 ± 0.34	0.70 ± 0.33	1.25 ± 0.79	0.041	0.063

Values are mean ± standard deviation. Tukey correction of *p* value for limbal nasal redness: pSS-DED vs. non-SS-DED *p* = 1.000, sSS-DED vs. pSS-DED *p* = 0.048, sSS-DED vs. non-SS-DED *p* = 0.189. *^a^* Reflects linear test for trend. *^b^* Ocular Surface Disease Index. *^c^*^,*d*^ First and average non-invasive keratography break-up time.

**Table 2:** The top five canonical pathways predicted in Ingenuity Pathway Analysis for differentially expressed proteins in sSS-DED patients versus pSS-DED patients.

Canonical Pathway	*p*-Value
Acute phase response signaling	4.93 × 10^−13^
LXR/RXR activation	1.69 × 10^−9^
FXR/RXR activation	1.95 × 10^−9^
Coagulation system	1.18 × 10^−5^
Complement system	1.33 × 10^−5^

**Table 3:** The top 5 canonical pathways predicted in Ingenuity Pathway Analysis for differentially expressed genes in sSS-DED patients versus non-SS-DED patients.

Canonical Pathway	*p*-Value
EIF2 signaling	2.33 × 10^−9^
Coronavirus pathogenesis pathway	1.95 × 10^−5^
Atherosclerosis signaling	3.07 × 10^−5^
IL-15 signaling	5.53 × 10^−5^
Caveolar-mediated endocytosis signaling	8.59 × 10^−5^

**Table 4:** The verification of various proteins with ELISA in tears of patients.

	Non-SS-DED (n = 11)	pSS-DED (n = 12)	sSS-DED (n = 11)	*p* Value	*p* Value (Trend)
EGF	12.94 ± 7.13	3.32 ± 6.82	17.73 ± 18.76	0.023	0.361
ADAM10	19.60 ± 5.05	21.35 ± 8.19	38.92 ± 27.54	0.018	0.011
Albumin	5270.98 ± 9990.46	21,160.28 ± 30,812.56	50,489.62 ± 105,118.12	0.243	0.101
S100A8	89.70 ± 45.23	78.33 ± 41.13	136.67 ± 102.74	0.116	0.117

Tukey correction of *p* value: EGF: pSS-DED vs. non-SS-DED *p* = 0.154, sSS-DED vs. pSS-DED *p* = 0.020, sSS-DED vs. non-SS-DED *p* = 0.627. ADAM10: pSS-DED vs. non-SS-DED *p* = 0.966, sSS-DED vs. pSS-DED *p* = 0.043, sSS-DED vs. non-SS-DED *p* = 0.028. Albumin: pSS-DED vs. non-SS-DED *p* = 0.807, sSS-DED vs. pSS-DED *p* = 0.505, sSS-DED vs. non-SS-DED *p* = 0.221. S100A8: pSS-DED vs. non-SS-DED *p* = 0.916, sSS-DED vs. pSS-DED *p* = 0.118, sSS-DED vs. non-SS-DED *p* = 0.255.

## Data Availability

The datasets generated and analyzed during the current study are available from the corresponding author on reasonable request.
